# Dexmedetomidine Attenuates Bilirubin-Induced Lung Alveolar Epithelial Cell Death In Vitro and In Vivo*

**DOI:** 10.1097/CCM.0000000000001035

**Published:** 2015-08-14

**Authors:** Jian Cui, Hailin Zhao, Bin Yi, Jing Zeng, Kaizhi Lu, Daqing Ma

**Affiliations:** 1 Department of Anesthesia, Southwest Hospital, Third Military Medical University, Chongqing, China.; 2 Section of Anesthetics, Pain Medicine and Intensive Care, Department of Surgery and Cancer, Faculty of Medicine, Imperial College London, Chelsea and Westminster Hospital, London, United Kingdom.

**Keywords:** anesthetics, hypoxia, liver disease, lung damage, lung edema

## Abstract

**Objective:**

To investigate bilirubin-induced lung alveolar epithelial cell injury together with the protection afforded by dexmedetomidine.

**Design:**

Prospective, randomized, controlled study.

**Setting:**

Research laboratory.

**Subjects:**

Sprague Dawley rats.

**Interventions:**

Alveolar epithelial A549 cell lines were cultured and received bilirubin (from 0 to 160 μM) to explore the protective pathway of dexmedetomidine on bilirubin-induced alveolar epithelial cell injury assessed by immunochemistry and flow cytometry. Sprague-Dawley rats were subjected to common bile duct ligation surgery to explore the protective effect of dexmedetomidine on hyperbilirubinemia-induced alveolar epithelial cell injury and respiratory failure in comparison with the Sham (subjected to the surgery procedure but without bile duct ligation) or dexmedetomidine control (only received intraperitoneal injection of dexmedetomidine).

**Measurements and Main Results:**

In vitro, dexmedetomidine reversed the collapse of mitochondrial membrane potential (Δψm), upregulation of cytochrome *C*, B cell leukemia 2 associated X protein, and cleaved-caspase 3 and 9 in A549 epithelial cells with bilirubin challenge. Furthermore, dexmedetomidine reversed the arrest of cell cycle and the downregulation of the transforming growth factorβ, phosphorylated mammalian target of rapamycin, and p42/44 mitogen-activated protein kinase induced by bilirubin. In vivo, pulmonary edema and inflammation were found after common bile duct ligation. Bilirubin and Paco_2_ were significantly increased, and oxygen (Pao_2_) was significantly decreased in the blood of common bile duct ligation rats from the postsurgery day 7 to day 21 when compared with those in the sham controls, respectively (*p* < 0.01). Daily intraperitoneal injection of dexmedetomidine significantly alleviated the lung edema and injury and prevented respiratory failure.

**Conclusion:**

Our data both in vitro and in vivo demonstrated that dexmedetomidine protected alveolar epithelial cell from bilirubin-induced injury. Dexmedetomidine may be a good choice of anesthetic/sedative for patients with chronic liver disease during the perioperative period.

Hepatopulmonary syndrome (HPS) is a serious complication frequently observed in patients with chronic liver disease (1, 2). Previous studies have shown that pathological changes occurring in the lungs of animals with HPS included nonspecific pneumonia and epithelial cells injury (3). The tidal volume, minute ventilation, and mean inspiratory flow were significantly decreased, and chest wall pressure dissipation against the resistive and viscoelastic components and elasticity were reduced (4). All of these changes result in ventilation-perfusion (V/Q) mismatch, diffusion limitation of oxygen, and, less commonly, arteriovenous shunt. Orthotropic liver transplantation was considered the favorable method to improve the survival rate of patients with HPS (5). Many other operations have been performed on patients with HPS in clinics to improve hepatic function, such as endoscopic sphincterotomy, multiple bile duct stone extraction, and hilar tumor resection.

Hyperbilirubinemia is the typical feature of most severe liver disease and was considered the main cause of HPS (6). Previous studies have identified that high concentration of bilirubin leads to release of inflammatory cytokines from glial cells or neuronal cell apoptosis in the brain (7). In the lung, pulmonary vascular dilatation and high permeability of the pulmonary vascular barrier were found in rats undergoing common bile duct ligation (CBDL) surgery (8). Following destruction of the vascular endothelial barrier, high concentrations of bilirubin can enter interstitial tissue and, therefore, could be in direct contact with pulmonary alveolar epithelial cells, but the potential effect of bilirubin on the lung epithelial cells remains incompletely understood.

Dexmedetomidine, a potent α_2_ adrenergic agonist, has been commonly used in operating room and ICU for its sparing effect of other anesthetics or sedation (9, 10). Recent studies have demonstrated that dexmedetomidine could reduce systemic inflammation in animals and improves the gas exchange in patients (11, 12). An elegant previous study has shown that dexmedetomidine confers a remote lung acute injury following kidney ischemia reperfusion injury in mice (13). In addition, it has been found that dexmedetomidine has potent renoprotective effects against renal ischemia reperfusion injuries, which is very likely associated with p-Akt and Janus kinase/signal transducer and activator of transcription signaling activation in mice and rats (14, 15).Taken together, these studies indicate that dexmedetomidine possesses cytoprotective effects. Thus, we hypothesized that dexmedetomidine might alleviate the epithelial cell injury in HPS. In this study, we aim to investigate whether dexmedetomidine protects the lung alveolar epithelium against injury associated with hyperbilirubinemia both in vivo and in vitro.

## MATERIAL AND METHODS

### Cell Culture and Treatments

The human alveolar epithelial cell line A549 (European Cell Culture Collection, Public Health English, Porton Down, Salisbury, UK) was used for this study. A549 cells were cultured in Roswell Park Memorial Institute 1640 (RPMI 1640) medium supplemented with 10% fetal bovine serum and cultured at 37° in 5% Co_2_. Cells were challenged with a gradient concentration of bilirubin (from 0 to 160 μM) in fresh RPMI 1640 medium free from fetal bovine serum for 24 hours. The concentration of bilirubin was chosen based on the blood test of unconjugated bilirubin of CBDL rats (16). Live cells counted from 10 random microscopic fields from each 6-cm culture dishes were analyzed. The average cell survival ratio relative to the naïve controls (NCs) was used for further data analysis. Dexmedetomidine concentrations were chosen from our previous study (14, 15), and the dose of atipamezole, an α_2_ adrenergic antagonist, was based on a binding affinity ratio 10:1 for agonist-antagonist (17). Briefly, the cells were pretreated with 1 nM dexmedetomidine for 15 minutes first in the presence or absence of 10 nM atipamezole and then exposed to 80 μM bilirubin for additional 24 hours. Cells were grouped as NC: no drug challenge; bilirubin alone (B80): 80 μM bilirubin; dexmedetomidine: 1 nM dexmedetomidine; dexmedetomidine + bilirubin (DB): pretreat with 1 nM dexmedetomidine for 15 minutes and then expose to 80 μM bilirubin; atipamezole: 10 nM atipamezole; atipamezole + dexmedetomidine + bilirubin: pretreat with atipamezole for 15 minutes and then treated with dexmedetomidine for another 15 minutes, then exposed to 80 μM bilirubin.

### Determination of Δψm In Vitro

A549 cells were labeled with lipophilic cationic probe 5, 5′, 6,6′-tetrachloro-1,1′,3,3′-tetraethylbenzimidazolcarbocyanineiodide (JC-1) and detected by flow cytometry and microscope separately (18, 19). Briefly, for flow cytometry detection, cells were detached using 0.25% trypsin and then transferred to 5 mL polystyrene tubes. After washing with fluorescence-activated cell sorting (FACS) buffer (10% fetal calf serum, 0.5 M EDTA in 0.1 M phosphate-buffered saline [PBS]) once, the cells were incubated with 0.2 μM JC-1 in FACS for 30 minutes at 37°C and protected from light. The cells were analyzed by flow cytometry (FACS Calibur; Becton Dickinson, Sunnyvale, CA) after washing with warm FACS buffer twice. Fluorescence was measured in the FL-1 (green fluorescence) and FL-2 (red fluorescence) channels, gating only on live cells. To detect the direct fluorescence changes of JC-1 staining, the cells cultured on cover slips were stained with JC-1 directly as previously stated with some modifications (19). Briefly, the cells were incubated with 10 μM JC-1 in FACS buffer for 30 minutes at 37°C after washing with warm 0.1 M PBS once. The cells were washed with warm 0.1 M PBS three times for 5 minutes each while carefully protecting from potent light, and then, the nuclei were stained using 4,6-diamidino-2-phenylindole (DAPI). The fluorescence stained on the cells was examined under rhodamine (red), fluorescein (green), and cyan (blue) spectral filter with Olympus (Watford, United Kingdom) BX40 microphotography system. The geometric mean of red fluorescence and green fluorescence of JC-1 staining on the cells was analyzed using FlowJo 7.6.1 software (TreeStar, San Carlos, CA) from eight independent flow cytometry detections. The mean ratio of red/green was calculated as an indicator of Δψm.

### Immunohistochemistry

For in vitro fluorescence staining, cells were fixed in paraformaldehyde in 0.1 M PBS solution. Cells were then incubated in 10% normal donkey serum and then incubated overnight with Rabbit anticytochrome *C* or B cell leukemia 2 associated X protein, B cell leukemia-2, cleaved-caspase 3 and 9, transforming growth factorβ (TGFβ), phosphorylated mammalian target of rapamycin (p-mTOR), and p42/44 mitogen-activated protein kinase (MAPK) (1:200; Santa Cruz, Dallas, TX) followed by secondary antibody for 1 hour. For in vivo fluorescence staining, 5-mm-thick paraffin sections were first dewaxed and subjected to heat-mediated antigen retrieval. Sections were incubated with donkey serum followed by the cleaved-caspase 3 antibody (1:200; Santa Cruz). After washing with PBS-Tween 20, the slides were incubated with fluorochrome-conjugated secondary antibodies (Millipore, Beeston, United Kingdom) for 1 hour. The slides were counterstained with nuclear dye DAPI and then examined by using an Olympus BX40 microscope under constant exposure level. Immunofluorescence was quantified using ImageJ (National Institutes of Health, Bethesda, MD), and the background was subtracted. Ten representative fields were randomly selected by an assessor blinded to the treatment groups. Values were then calculated as percentages of the mean value for NCs and expressed as percentage fluorescence. The proportion of positive cells was calculated as the number of positive cells relative to the number of DAPI-positive cells.

### Cell Cycle Analysis by Flow Cytometry

The cell cycle was analyzed by flow cytometry as described previously (20). The cells were detached from the 24-well culture plate with 0.25% trypsin and then transferred to 5 mL polystyrene tubes specifically designed for flow cytometry. After washing twice with 0.1 M PBS, the cells were fixed with 70% ethanol at 4° overnight. After centrifuging at 2,500 rpm for 10 minutes and resuspending in 500 μL freshly prepared FACS buffer, 10 μL of 40 μg/L propidium iodide (PI) and 10 μL of 500 ng/L ribonuclease were added to the cell suspension and kept in a dark place for 10 minutes. Fluorescence of PI stained on the cells was detected with flow cytometry and analyzed with FlowJo 7.6.1 software (FACS Calibur, Becton Dickinson, Sunnyvale, CA). For the cell cycle analysis, a minimum of 10,000 cells per sample were analyzed with flow cytometry (TreeStar, San Carlos, CA; BioRad, Hemel Hempstead, UK). Data were analyzed by FlowJo software (TreeStar; BioRad), which showed basic statistics such as the fraction of cells in G0/1, S, and G2, the positions of the G0/1 and G2 peaks, and their widths. The percentage of cells in different phases of the cell cycle was therefore determined.

### Animals and Surgical Procedure

This study was approved by the Ethics Committee of Animal Experiments of Third Military Medical University. Every effort was made to minimize animal suffering and the number of animals used. Sprague-Dawley rats (220–250 g) were used for experiments and were kept under a 12-hour light/dark cycle with free access to food and water. Hyperbilirubinemia was induced by modified CBDL as we reported before (21, 22). Aseptic laparotomy was made in Sprague-Dawley rats (220–250 g) under 3.5% chloral hydrate anesthesia (10 mL/kg, IP). The common bile duct was identified and double ligated with 4-0 cotton sutures (CBDL). Just laparotomy without bile duct ligation or without any surgery served as the Sham controls and NCs, respectively. They were allowed to recover in individual cages as reported previously (13, 14); 25 μg/kg dexmedetomidine or the same volume saline (as vehicle control) was intraperitoneal (IP) injected after 3 hours of CBDL surgery on the 1st day and then for 6 consecutive days. Dexmedetomidine-controlled rats only received IP injection of 25 μg/kg dexmedetomidine daily and without any surgery. At the end of the experiments, the rats received terminal anesthesia (chloral hydrate 350 mg/kg, IP), and 2 mL blood was immediately collected through a needle punctured in left ventricle of heart. Blood gas and unconjugated bilirubin were measured with routine clinical laboratory apparatuses. The lungs were, subsequently, perfused with 4% paraformaldehyde under constant pressure and then embedded into paraffin and sectioned into 5 μm for further histological analysis.

### Histological Analysis

The sections were stained with hematoxylin and eosin (H&E) staining, and the morphology in each lung (10 fields at ×20 magnifications) was evaluated by an observer who was blinded to the treatments under an Olympus BX40 microscope. The score for each field was calculated from the sum score of 10 areas chosen randomly. The injury of the lung alveolar epithelial cells was categorized (23) into grade 0: normal appearance, negligible damage; grade 1: mild-moderate interstitial congestion and neutrophil leukocyte infiltrations; grade 2: perivascular edema formation, partial destruction of pulmonary architecture, and moderate cell infiltration; grade 3: moderate lung alveolar damage and intensive cell infiltration; grade 4: severe cell infiltration and severe destruction of the pulmonary architecture.

### Lung Wet/Dry Ratio

The lungs harvested from other cohorts after experiments under terminal anesthesia were weighed to obtain wet weight and then dried for 48 hours at 80°C oven to obtain the dry weight. The wet-to-dry ratio was calculated as an indicator of pulmonary edema.

### Western Blotting

Lung samples were mechanically homogenized in lysis buffer. The cell lysates were centrifuged and then supernatant was collected, and total protein concentration in the supernatant was quantified by the Bradford protein assay (BioRad). The protein extracts (40 μg/sample) were heated, denatured, and loaded on a NuPAGE 4–12% Bis-Tris gel (Invitrogen, Paisley, UK) for electrophoresis and then transferred to a polyvinylidene difluoride membrane. The membrane was treated with blocking solution (5% dry milk in Tris buffered saline with 0.1% Tween-20) for 2 hours and probed with cleaved-caspase 3 antibody (1:1,000; Santa Cruz) overnight at 4°C, followed by horseradish peroxidase-conjugated secondary antibody for 1 hour. The loading control was the constitutively expressed protein β-actin (1:10,000; Sigma-Aldrich, St. Louis, MO). The blots were visualized with enhanced chemiluminescence system (Santa Cruz) and analyzed with GeneSnap (Syngene, Cambridge, United Kingdom).

### Terminal Deoxynucleotidyl Transferase–Mediated dUTP Nick-End Labeling Assay

Lung alveolar cell apoptosis was detected using the one-step terminal deoxynucleotidyl transferase dUTP nick-end labeling (TUNEL) apoptosis assay kit (Beyotime, Shanghai, China) according to the manufacturer’s instructions. In brief, the fixed, paraffin-embedded sections were deparaffinized and washed with PBS, labeled with terminal-deoxynucleotidyl transferase enzyme at 37°C for 0.5 hour, and then incubated with 5-bromo-4-chloro-3-indolyl-phosphate substrate labeled with Cy-3 for 40 minutes and DAPI for 5 minutes at room temperature. Ten randomly chosen fields were examined under ×40 view, and the proportion of TUNEL-positive cells was calculated as the number of TUNEL-positive cells relative to the number of DAPI-positive cells.

### Statistical Analysis

All numerical data were presented as mean ± sd or otherwise box-whisker plot. Comparison between the treatment groups was analyzed by one-way analysis of variance followed by Tukey multiple group comparisons (equal variances) or Kruskal-Wallis (nonparametric) test followed by Dunn multiple group comparisons (unequal variances or scoring) (GraphPad Prism 5, San Diego, CA). A *p* value of 0.05 was considered as statistically significant.

## RESULTS

### Effects of Dexmedetomidine on Δψm, Cytochrome *C* Release in A549 Cells After Bilirubin Challenge

**Figure 1.**
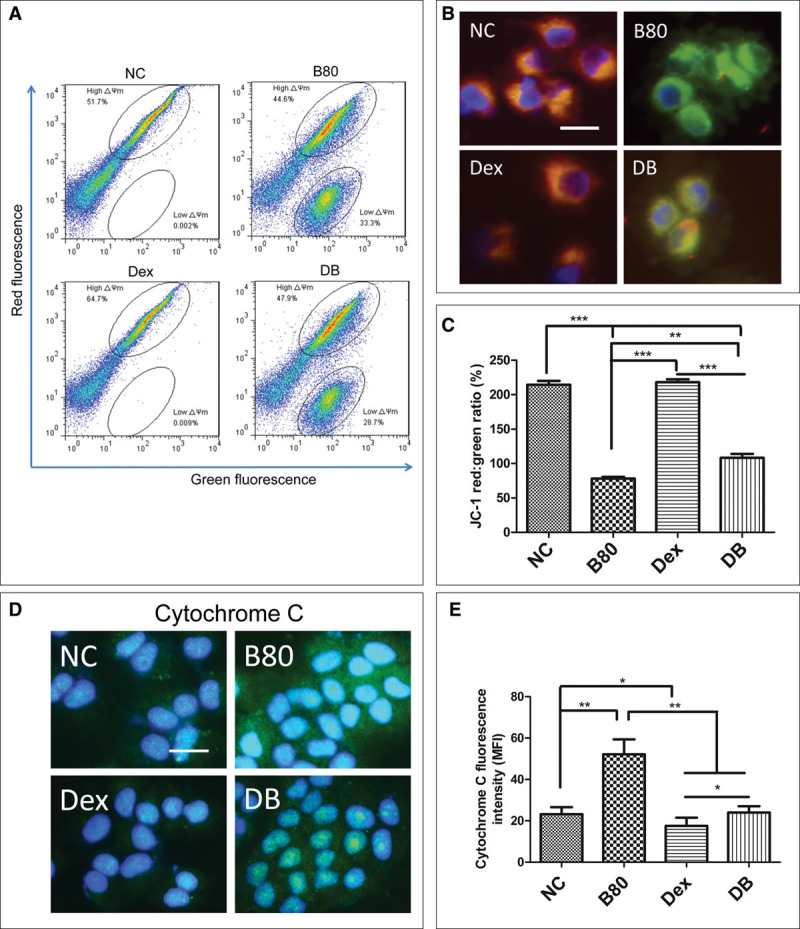
Effect of dexmedetomidine (Dex) on the bilirubin-induced Δψm collapse and release of cytochrome *C* from mitochondria in A549 cells. A549 cells were pretreated with 1 nM dexmedetomidine for 15 min and then exposed to 80 μM bilirubin for additional 24 hr. **A**, Δψm was assessed by flow cytometry under fluorescein isothiocyanate and phycoerythrin channel after stained with 5, 5′, 6, 6′-tetrachloro-1,1′,3,3′-tetraethylbenzimidazolcarbocyanine iodide (JC-1). **B**, A549 cells were stained with JC-1, assessed through immunofluorescence microscope. Nuclear was counter stained with 4,6-diamidino-2-phenylindole. **C**, JC-1 fluorescence intensity ratio (*red*/*green*) compared with each other was from data of eight independent experiments of flow cytometry. **D**, Expression of cytochrome *C* in A549 cells detected with immunofluorescence. **E**, Fluorescence intensity of cytochrome *C*. Data are mean ± sd. *n* = 8. *p < 0.05 and **p< 0.01 and ***p < 0.001, scale bar: 50 μm. B80 = final concentration of bilirubin was 80 μM, MFI = mean fluorescence intensity, NC = naïve controls.

Δψm was measured with flow cytometry and microscope separately after JC-1 staining. In cells in NC and dexmedetomidine groups, the intensity of red fluorescence was about two times green (no significant difference in the ratio of red/green between these two groups; *p* > 0.05). In bilirubin challenged cells (the B80 group), there were 33.3% ± 2.71% cells presenting with low Δψm and this proportion of cells decreased to 28.7% ± 1.98% in the dexmedetomidine pretreated cells (DB group) (*p* < 0.01) (**Fig. 1*A***). Meanwhile, the mean ratio of red/green of all cells in the B80 group was 77.96 ± 5.18% and was increased to 107.74 ± 13.00% in the DB group (**Fig. 1*C***). Cytochrome *C* released from the mitochondria in A549 cells was detected by immunofluorescence. Enhanced expression of cytochrome *C* was found after exposure to 80 μM bilirubin for 24 hours (*p* < 0.01 compared to NC), which was partly reversed by 1 nM dexmedetomidine (*p* < 0.01) (**Fig. 1**, ***D*** and ***E***).

### Effects of Dexmedetomidine on BAX, BCL-2, and Cleaved-Caspase 3 and 9 Expression in A549 Cells After Being Challenged by Bilirubin

**Figure 2.**
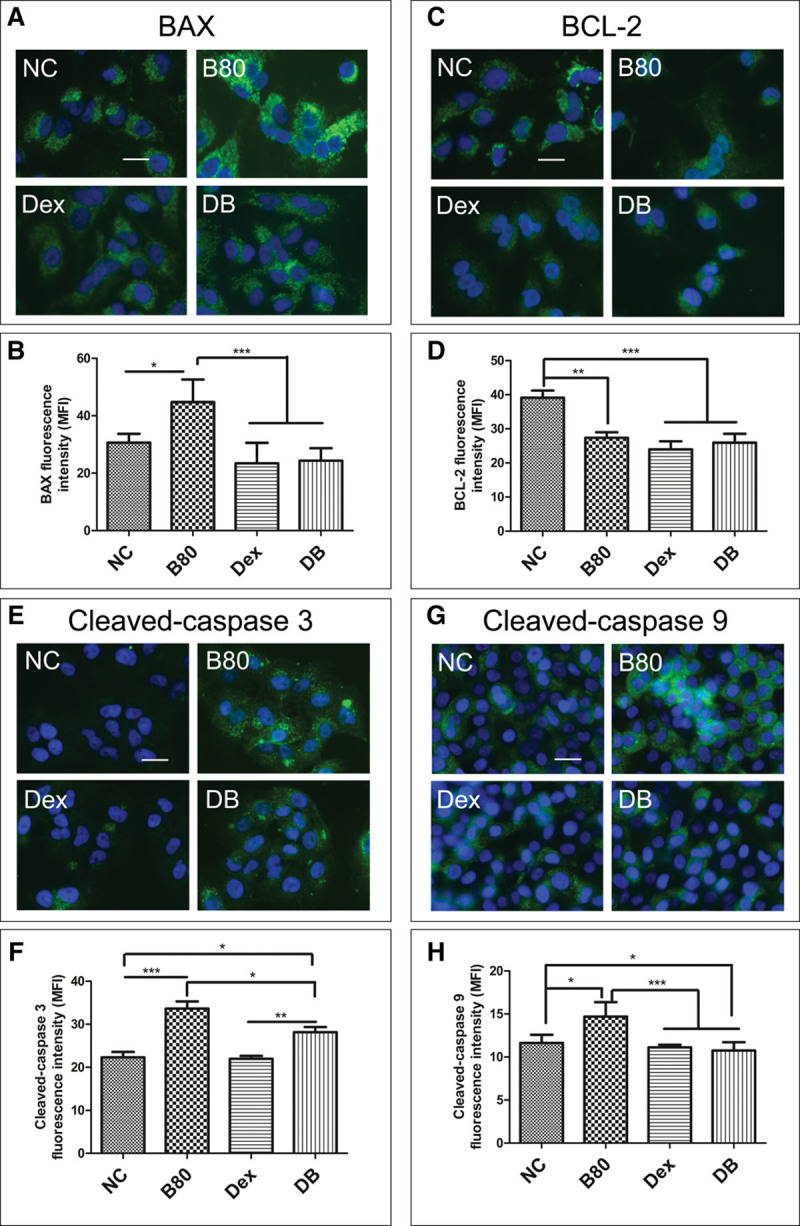
Dexmedetomidine (Dex) reversed the bilirubin-induced BAX, cleaved-caspase 3, and caspase 9 overexpression but had no effect on BCL-2 downregulation. A549 cells were challenged with 80 μM bilirubin for 24 hr with or without pretreatment with 1 nM dexmedetomidine. **A**, Green fluorescence showed the overexpression of BAX in the cells exposed to bilirubin and which was reversed by pretreat with dexmedetomidine. **B**, The mean fluorescence intensity of BAX increased in bilirubin-challenged A549 cells and which was downregulated by pretreat with 1 nM dexmedetomidine. **C**, The expression of BCL-2 (*green*) in A549 cells. **D**, BCL-2 expression in A549 cells after bilirubin challenge with or without dexmedetomidine pretreat. **E**, Green fluorescence showed the expression of cleaved-caspase 3 in A549 cells. Overexpression of cleaved-caspase 3 induced by bilirubin was inhibited by pretreat with dexmedetomidine; histogram of cleaved-caspase 3 fluorescence intensity (**F**). Dexmedetomidine inhibited the overexpression of cleaved-caspase 9 in A549 cells; green fluorescence showed the expression of cleaved-caspase 9 in A549 cells (**G**); histogram of cleaved-caspase 9 fluorescence intensity (**H**). Dexmedetomidine inhibited the overexpression of cleaved-caspase 9 in A549 cells. Data are mean ± sd. *n* = 8. *p < 0.05 and **p < 0.01 and ***p < 0.001, scale bar: 50 μm. B80 = final concentration of bilirubin was 80 μM, DB = dexmedetomidine + bilirubin, MFI = mean fluorescence intensity, NC = naïve controls.

Immunofluorescence study revealed dexmedetomidine significantly inhibited upregulation of BAX expression in A549 cells induced by bilirubin (*p* < 0.001) (**Fig. 2**, ***A*** and ***B***). Decreased fluorescence intensity of BCL-2 was found in bilirubin- and dexmedetomidine-treated A549 cells. No significant difference of mean fluorescence intensity (MFI) was found among bilirubin, dexmedetomidine, and combined treated cells (*p* > 0.05) (**Fig. 2**, ***C*** and ***D***). Upregulation of cleaved-caspase 3 and 9 expression in A549 cells was detected after 80 μM bilirubin treatment for 24 hours (*p* < 0.05 vs NC). Dexmedetomidine completely reversed the upregulation of cleaved-caspase 9 but only had a partial effect on that of cleaved-caspase 3 induced by bilirubin in A549 cells (**Fig. 2*E*–*H***).

### Effects of Dexmedetomidine on Activation of TGFβ, p-mTOR, and p44/42MAPK Pathways in A549 Cells After Being Challenged by Bilirubin

**Figure 3.**
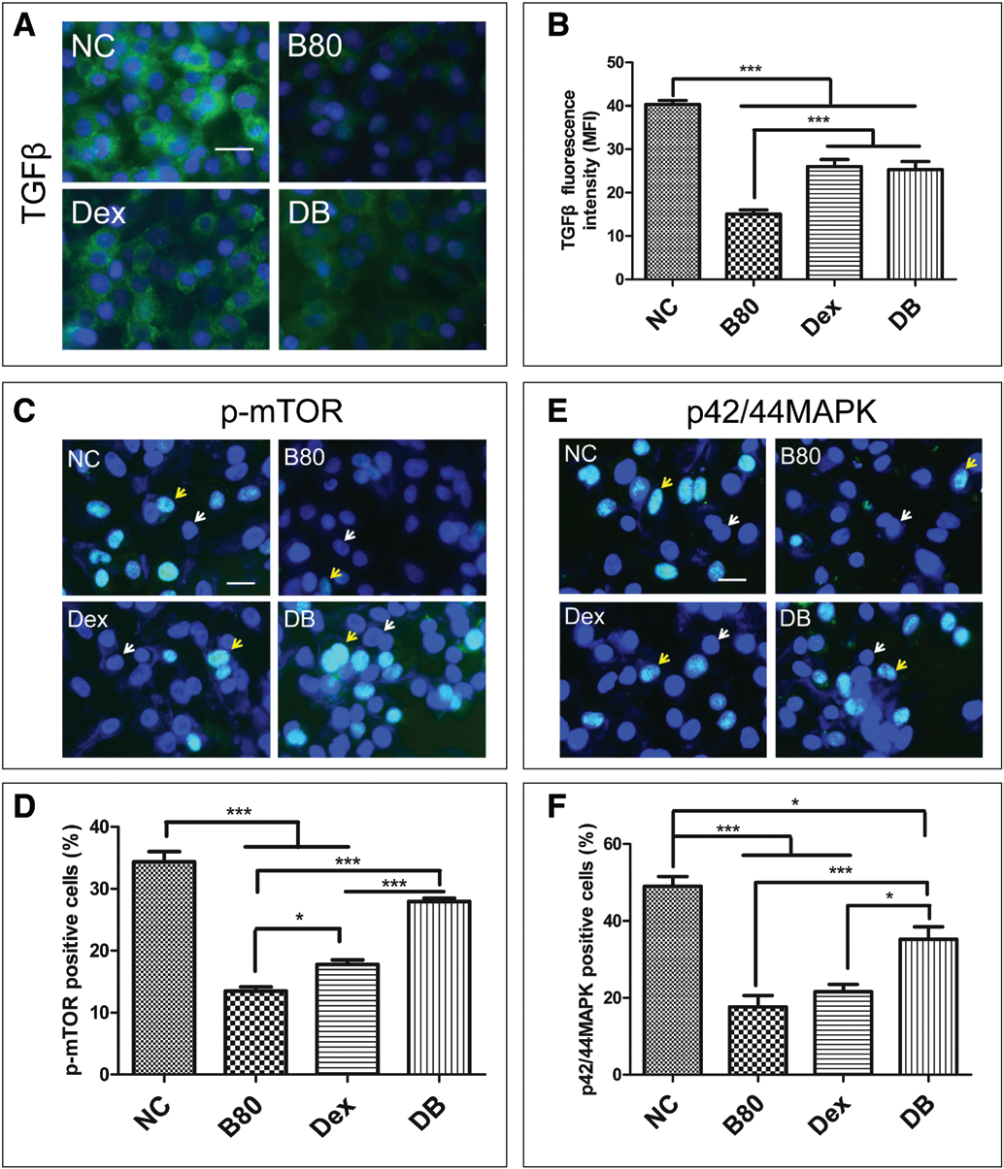
Dexmedetomidine (Dex) reversed the bilirubin-induced downregulation of TGFβ, phosphorylated mammalian target of rapamycin (p-mTOR), and p42/44 mitogen-activated protein kinase (MAPK) in A549 cells. A549 cells were challenged with 80 μM bilirubin for 24 hr with or without pretreatment with 1 nM dexmedetomidine. **A**, Expression of TGFβ (*green*) was assessed by immunofluorescence staining. Nuclear was counterstained with 4,6-diamidino-2-phenylindole (DAPI); the mean green fluorescence intensity of TGFβ (**B**); expression of p-mTOR (green fluorescence) in A549 cells was assessed by immunofluorescence staining (**C**). Nuclear was counterstained with DAPI; percentage of p-mTOR-positive cells (**D**); expression of p42/44MAPK (*green*) in A549 cells was assessed by immunofluorescence staining (**E**). Nuclear was counterstained with DAPI; the percentage of p42/44MAPK-positive cells (**F**). Data are mean ± sd. *n* =8. *p < 0.05, **p < 0.01, ***p < 0.001. Scale bar = 50 μm. *White arrow*, negative cells; *yellow arrow*, positive cells. B80 = final concentration of bilirubin was 80 μM, DB = dexmedetomidine + bilirubin, MFI = mean fluorescence intensity, NC = naïve controls.

TGFβ expression in A549 cells was determined by immunofluorescence to explore the protective effect of dexmedetomidine on bilirubin-induced cell apoptosis. The result indicated that 80 μM bilirubin and 1 nM dexmedetomidine significantly inhibited the expression of TGFβ (*p* < 0.001). Pretreatment with dexmedetomidine for 15 minutes partly reversed the downregulation of TGFβ expression induced by bilirubin (MFI, 15.06 ± 3.00 in B80 group vs 25.32 ± 5.94 in DB group; *p* < 0.001). There was no significant difference in MFI between dexmedetomidine and DB groups (*p* > 0.05) (**Fig. 3**, ***A*** and ***B***). The percentage of p-mTOR and p44/42MAPK-positive cells was reduced by either 80 μM bilirubin or 1 nM dexmedetomidine (*p* < 0.001 vs NC). The percentage of p-mTOR-positive cells increased from 13.25% ± 2.01% in the B80 group to 29.35% ± 2.96% in the DB group (*p* < 0.001). The percentage of p44/42MAPK-positive cells also increased in the dexmedetomidine pretreated cells (18.37% ± 6.51% in the B80 group vs 35.32% ± 7.28% in the DB group; *p* < 0.001) (**Fig. 3*C*–*F***).

### Effects of Dexmedetomidine on Cell Cycle Progression of A549 Cells Following Bilirubin Challenge

**Figure 4.**
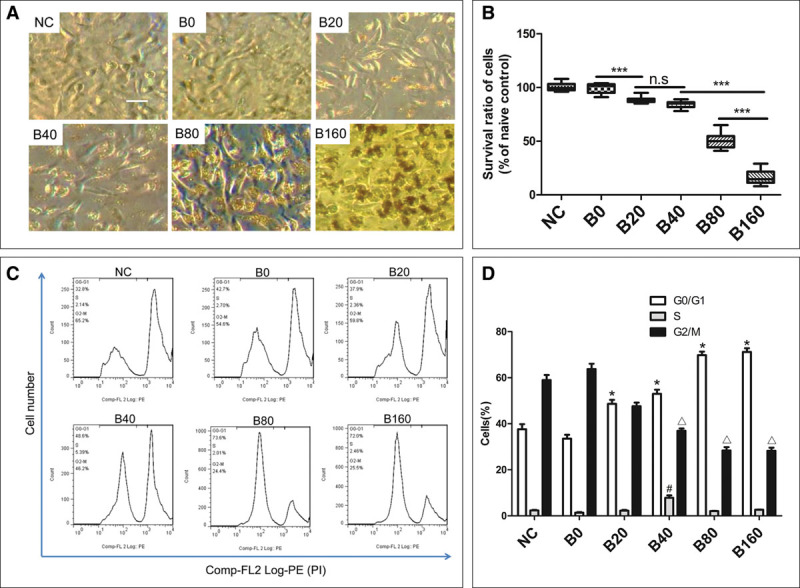
Bilirubin-induced cell death and arrest of G0/G1 phase of live A549 cells in a concentration-dependent manner. A549 cells were challenged with gradually increased concentration of bilirubin for 24 hr. **A**, Cell morphology of A549 cells after treated with gradually increased bilirubin for 24 hr. **B**, Cell survival ratios after being challenged with a gradient concentration of bilirubin. ***p < 0.001; n.s. = no significant difference. **C**, Cell cycle of A549 cells after bilirubin challenge, assessed by propidium iodide flow cytometry. **D**, Percentage of cells at G0/G1, S, and G2/M after bilirubin challenge. *p < 0.001 compared with the cells in G0/G1 phase of naïve control (NC); #*p* < 0.001 compared with the cells in S phase of NC; Δ*p* < 0.001 compared with the cells in G2/M phase of NC. B0 = final concentration of bilirubin was 0 μM, B20 = final concentration of bilirubin was 20 μM, B40 = final concentration of bilirubin was 40 μM, B80 = final concentration of bilirubin was 80 μM, B160 = final concentration of bilirubin was 160 μM. Data are mean ± sd. *n* = 8. *p < 0.05, **p < 0.01, ***p < 0.001. Scale bar = 100 μm.

**Figure 5.**
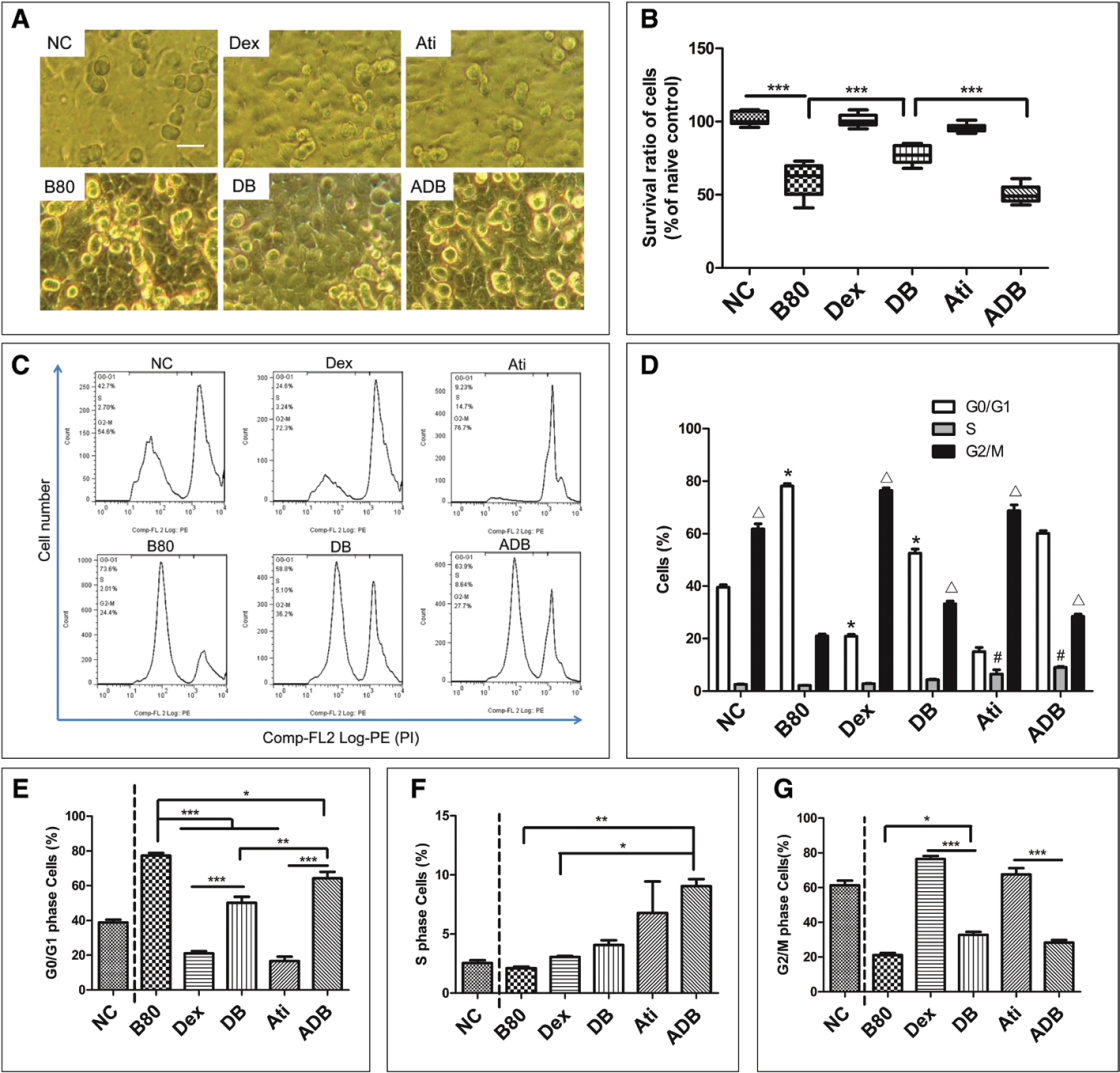
Dexmedetomidine (Dex) reversed the arrest of G0/G1 phase of A549 cells independent on α_2_ receptor. A549 cells were pretreated with 1 nM dexmedetomidine (or combined with its antagonist, 10 nM atipamezole [Ati]) for 15 min, then treated with 80 μM bilirubin for additional 24 hr. **A**, Dexmedetomidine attenuated the cell death induced by 80 μM bilirubin. Fewer cells dispatched from the bottom of plate in dexmedetomidine + bilirubin group cells compared with that in bilirubin treated only (B80 group). This effect was reversed by 10 nM atipamezole, scale bar = 100 μm. **B**, Summary of the cell survival ratio changes, ***p < 0.001. **C**, Dexmedetomidine reversed the G0/G1 arrest in A549 cells induced by 80 μM bilirubin, but it could not be completely reversed by the α_2_ antagonist (10 nM atipamezole). **D**, Statistical analysis of cell cycle after dexmedetomidine (associated with atipamezole or not) and bilirubin treatment and compared with that in naïve control (NC). *p < 0.001 compared with the cells in G0/G1 phase of NC; #*p* < 0.001 compared with the cells in S phase of NC; Δ*p* < 0.001 compared with the cells in G2/M phase of NC. **E**, Comparison among the treatment groups about the percent of G0/1 phase cells. (**F**, Comparison among the treatment groups about the percent of S phase cells. **G**, Comparison among the treatment groups about the percent of G2/M phase cells. Data are mean ± sd in bar graph E, F, and G. *n* = 8. *p < 0.05, **p < 0.01, ***p < 0.001. DB = dexmedetomidine + bilirubin, ADB = atipamezole + dexmedetomidine + bilirubin.

The concentration-dependent effect of bilirubin on cell morphology and survivability of A549 cells was observed under the microscope. Bilirubin induced a concentration-dependent decrease of cell survival ratio after 24-hour treatment (**Fig. 4**, ***A*** and ***B***). The cell cycle of A549 cells were investigated using flow cytometry. The percentage of the cells in G0/G1 phase gradually increased with the increasing of bilirubin concentration. There was no significant difference in S phase cells detected between each group, except B40 (*p* < 0.001 vs NC), and accordingly, the percentage of cells in G2/M phase decreased gradually (**Fig. 4**, ***C*** and ***D***). Dexmedetomidine at 1 nM concentration was used to explore its effect on 80 μM bilirubin-induced cell death and G0/G1 arrest. Dexmedetomidine was found to increase the survivability of the A549 cells after bilirubin challenge. Blockage of the conjugation between dexmedetomidine and α_2_-adrenoceptor with high concentration of atipamezole (10 nM) partially abolished the effect of dexmedetomidine (**Fig. 5**, ***A*** and ***B***). The percentage increase in G0/G1 and decrease in G2/M of cell cycle was found after A549 cells being challenged with 80 μM bilirubin and which was partly revised by pretreatment with 1 nM dexmedetomidine (from 77.35% ± 3.04% decreased to 51.73% ± 4.79% in G0/G1 phase and from 20.17% ± 2.89% increased to 36.22% ± 3.12% in G2/M phase; *p* < 0.05). There was no significant difference about the S phase cells between B80 and DB groups (*p* > 0.05). The revising effect of dexmedetomidine on bilirubin-induced G0/G1 phase percentage increase could be partly abolished by 10 nM atipamezole (from 51.73% ± 4.79% to 59.28% ± 3.92%; *p* < 0.01) (**Fig. 5*C*–*G***).

### CBDL Operation Induced Lung Edema, Alveolar Epithelial Cell Apoptosis, and Respiratory Failure, Which Were Attenuated by Dexmedetomidine

**Figure 6.**
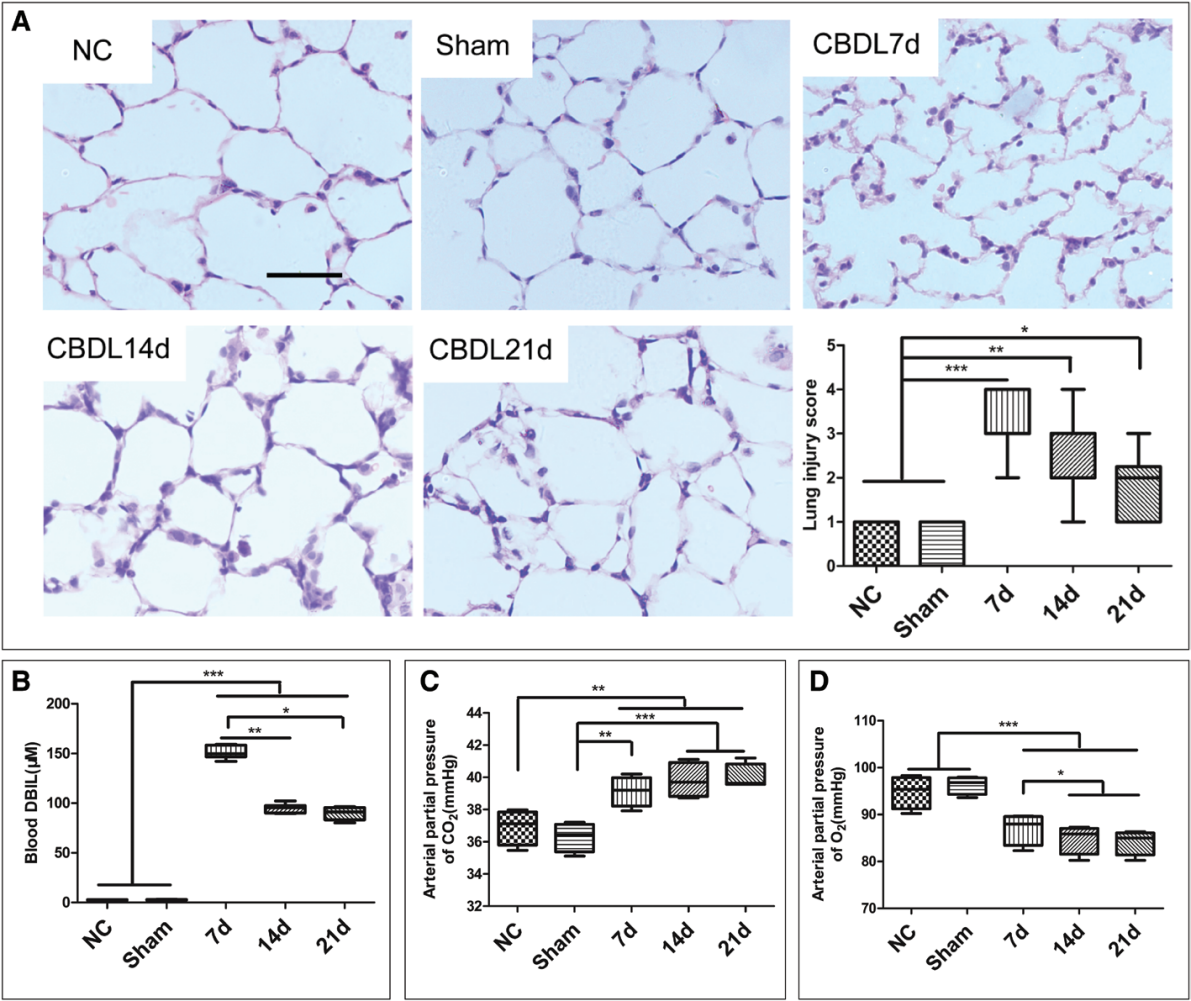
Common bile duct ligation (CBDL) surgery induced a persistent hyperbilirubinemia and pulmonary injury in Sprague Dawley (SD) rats. SD rats were subjected to CBDL operation, and the blood and lung samples were harvested at 7, 14, and 21 d after operation. Just laparotomy without bile duct ligation or without any surgery served as the sham controls and naïve controls (NCs), respectively. **A**, Histology assessment of lung in rats after CBDL surgery. **B**, Blood direct bilirubin (DBIL) (also known as unconjugated bilirubin) concentration increased in SD rats. Blood DBIL concentration increased to a very high level at day 7 after CBDL operation and remained at relative high level at days 14 and 21 (*p* < 0.001 relative to sham and NC). **C**, Pao_2_ gradually decreased after CBDL operation on SD rats. **D**, Paco_2_ gradually increased after CBDL operation on SD rats. n.s. = no significant difference. *n* = 10, *p < 0.05, **p < 0.01, ***p < 0.001. Scale bar = 50 μm.

**Figure 7.**
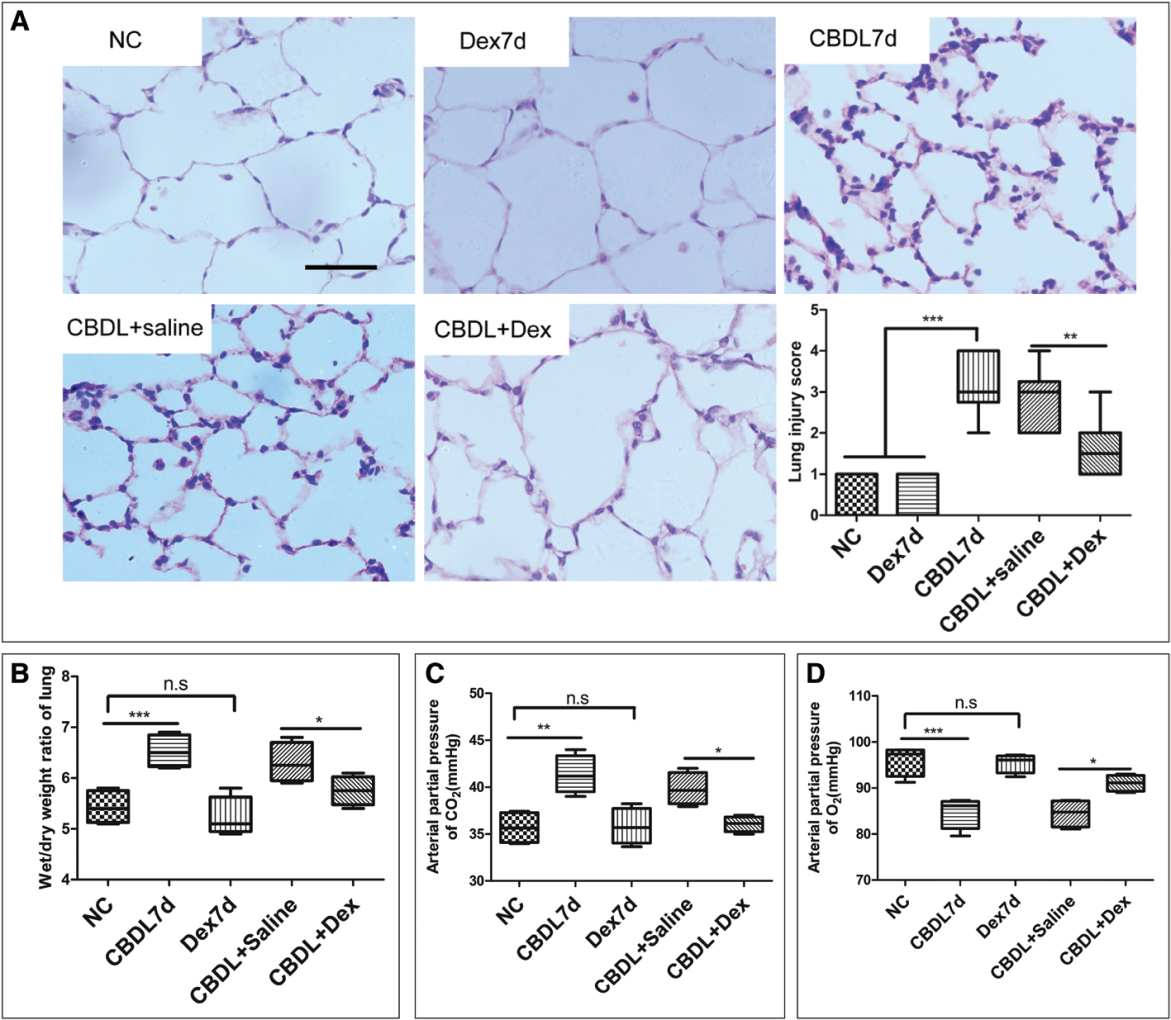
Intraperitoneal injection of dexmedetomidine (Dex) attenuated the pulmonary injury and respiratory failure of common bile duct ligation (CBDL) rats. A 25 μg/kg dexmedetomidine or the same volume saline (as vehicle control) was administered (intraperitoneally, IP) daily for 7 d after CBDL surgery. Dexmedetomidine-controlled rats only received IP injection of 25 μg/kg dexmedetomidine daily and without any surgery for 7 d. Hematoxylin and eosin staining of the lung sample (**A**); wet-to-dry ratio in lung samples of CBDL rats (**B**); decrease of Pao_2_ in CBDL rats was attenuated by dexmedetomidine on the day 7 after CBDL operation (**C**); increase of Paco_2_ in CBDL rats was inhibited by dexmedetomidine on the day 7 after CBDL operation (**D**). Dex7d = dexmedetomidine control rats, rats received injection of 25 μg/kg dexmedetomidine intraperitoneally for 7 d, NC = naïve control, n.s. = no significant difference. *n* = 10, *p < 0.05, **p < 0.01, ***p < 0.001. Scale bar = 50 μm.

**Figure 8.**
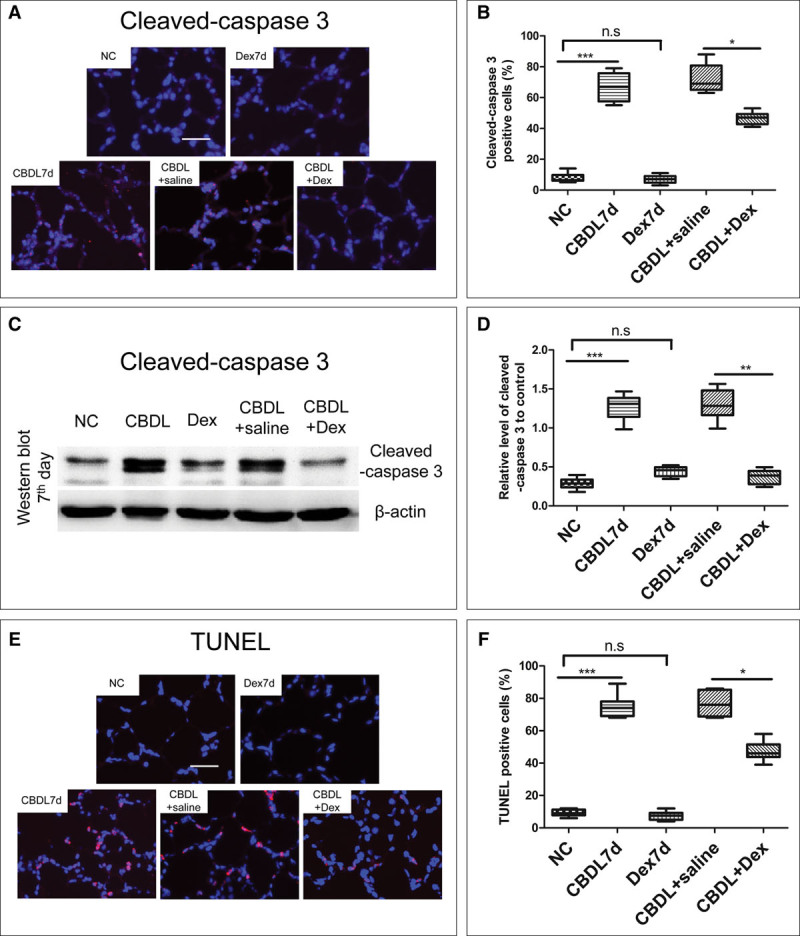
Dexmedetomidine (Dex) attenuated the apoptosis of lung cells of the common bile duct ligation (CBDL) rats after 7 d of operation. A 25 μg/kg dexmedetomidine or the same volume saline (as vehicle control) was administered (IP) daily for 7 d after CBDL surgery. Samples of lungs were harvested for immunochemistry and terminal deoxynucleotidyl transferase dUTP nick-end labeling (TUNEL) assay. **A**, Expression of cleaved-caspase 3 in the lungs of controlled and 7th day CBDL rats detected using immunochemistry. **B**, Percentage of cleaved-caspase 3 positive staining cells in rat lungs. **C**, Expression of cleaved-caspase 3 in the lungs of control and 7th day CBDL rats detected using western blotting. **D**, Expression of cleaved-caspase 3 in the lungs of control and 7th day CBDL rats. **E**, TUNEL-positive cells in the lungs of control and 7th day CBDL rats. **F**, Percentage of TUNEL-positive cells in the lungs. *n* = 10, ***p < 0.001. NC = naïve control, n.s. = no significant difference. Scale bar = 50 μm.

All rats survived until euthanize. After CBDL operation, the lung alveolar epithelial cells were flattened and the size of alveoli became more variable compared with naïve and sham rats when observed with H&E staining and the lung injury score increased significantly when compared with that in naïve or sham control rats (*p* < 0.001) (**Fig. 6*A***). The level of blood unconjugated bilirubin increased significantly on the 7th day and remained at a relatively higher level on the 14th and 21st day (remained at 80.31–156.71 μM; *p* < 0.001 vs sham). The Pao_2_ of CBDL rats decreased gradually as the time extension and adverse tendency was observed on the Paco_2_ (**Fig. 6, *B*–*D***). Compared with the naïve and dexmedetomidine control rats, the pulmonary edema and inflammation were significantly reduced in those dexmedetomidine-treated CBDL rats when observed with H&E staining at day 7 after operation. Meanwhile, IP injection of dexmedetomidine attenuated the increased ratio of lung wet/dry weight, increased blood Paco_2_ and decreased blood Pao_2_ in CBDL rats (*p* < 0.05, compared with CBDL rats and saline-treated CBDL rats) (**Fig. 7*A*–*D***). Cleaved-caspase 3 expression in the lungs of CBDL rats at day 7 after operation was significantly attenuated by dexmedetomidine when detected with both immunochemistry and western blotting (*p* < 0.05, compared with CBDL rats and saline-treated CBDL rats) (**Fig. 8*A*–*D***). Dexmedetomidine also inhibited the increase of TUNEL-positive cells in the lungs of CBDL rats at day 7 after operation (*p* < 0.05, compared with CBDL rats and saline-treated CBDL rats) (**Fig. 8**, ***E*** and ***F***).

## DISCUSSION

The present study, for the first time, explored the effect of dexmedetomidine, an α_2_ receptor agonist, on bilirubin-induced lung alveolar epithelial cell injury both in vivo and in vitro. Our results demonstrated that 1) cell death and G0/G1 cell cycle arrest of A549 cells were detected after bilirubin treatment for 24 hours in a concentration-dependent manner; 2) dexmedetomidine attenuated bilirubin-induced cell death and enhanced the proliferation of alveolar epithelial cells; 3) persistent hyperbilirubinemia was observed after CBDL operation, which may contribute to the lung alveolar epithelial injury and respiratory failure in rats; and 4) dexmedetomidine attenuated the pulmonary epithelial cell injury and respiratory failure of CBDL rats.

In our study, bilirubin at high concentrations induced epithelial cell apoptosis albeit in vitro. This is in line with a previous study in which bilirubin induced rat brain neuronal apoptosis via the mitochondrial pathway (24). Mitochondrial dysfunction has been shown to be involved in the induction of apoptosis (18). The opening of the mitochondrial permeability transition pore has been demonstrated to induce the depolarization of Δψm, release of apoptogenic factors such as cytochrome *C*, and disruption of adenosine triphosphate production (25). Mounting evidence indicates that disruption of the Δψm initiates the caspase cascade, leading to downstream activation of apoptosis (26, 27). Proapoptotic molecule BAX is essential in mitochondrial apoptotic pathway (28). In healthy mammalian cells, BAX is essentially cytosolic and inactive. Following a death signal, BAX protein is translocated to the outer mitochondrial membrane, where it promotes a permeabilization that favors the release of many apoptogenic factors, such as cytochrome *C*. Furthermore, BAX is a main member of the BCL-2 proapoptotic protein family. BCL-2, another member of BCL-2 family, counteracts BAX in the permeabilization of outer mitochondrial membrane and cell apoptosis (29). Increased BAX/BCL-2 ratio contributes to the initiation of caspase-induced apoptosis (30). In this study, bilirubin upregulated BAX and downregulated BCL-2 in A549 cells; in addition, dexmedetomidine treatment reversed the overexpression of BAX. Although dexmedetomidine did not stop the downregulation of BCL-2, the ratio of BAX/BCL-2 increased significantly (Fig. 2 *A*–*D*). Overexpression of initiator cleaved-caspase 9 and executioner cleaved-caspase 3 after bilirubin treatment was observed, which was also inhibited by pretreatment with dexmedetomidine (Fig. 2 *E*–*H*). These results indicated dexmedetomidine inhibited caspase-executed cell apoptosis not only at the early stage but also in the late phase.

TGFβ has been shown to play a key role in tissue repair after damage (31). TGFβ regulate protein phosphorylation in the cytoplasm and then transfer the proliferation signal to mitochondria (32). In this study, TGFβ was found to be downregulated after bilirubin treatment, which could be partially reversed by dexmedetomidine (Fig. 3, *A* and *B*). These results indicated that dexmedetomidine protected the epithelial cells from the apoptotic lesion through reversing the inhibition of TGFβ, which may facilitate the cell proliferation. Both p-mTOR and p42/44MAPK were found to be downregulated after bilirubin treatment, which could be reversed by pretreatment with dexmedetomidine (Fig. 4 *C*–*F*). Increasing evidence shows that mammalian target of rapamycin (mTOR) plays an important role in TGFβ-mediated cell proliferation, protein phosphorylation, and epithelial mesenchymal transition (33, 34). The downregulated p-mTOR indicated that proliferation of cells was inhibited, which was considered to contribute to the repair deficiency of alveoli after the insult of bilirubin. p42/44MAPK contributed to the proliferation of cells, dependent on mitochondria, just like mTOR (35, 36). Dexmedetomidine recovered bilirubin-induced inhibition of p-mTOR and p42/44MAPK indicated that dexmedetomidine may be helpful to repair the injured alveoli after bilirubin insult through increasing the proliferation of epithelial cells. Although the molecular mechanisms of bilirubin-induced lung cell injury is far more complicated, some molecular entities that may be responsible together with the interaction of dexmedetomidine have been found in our study but warrant further studies.

HPS was reported to be present in 4–32% of adult patients with end-stage liver disease (37). HPS was characterized as intrapulmonary vascular dilatation and arterial hypoxemia. A high concentration of bilirubin was considered to cause HPS. In this study, HPS induced by hyperbilirubinemia via CBDL in rats. It is a widely accepted model in this area of research (38). Indeed, it was found in this study that damage to lung alveoli was aggravated following the increase in blood bilirubin concentration. Meanwhile, Paco_2_ was increased and Pao_2_ was decreased gradually after CBDL operation, and all of these indicated that high concentration bilirubin is very likely contributes to the injury of lung alveolar epithelial cells and respiratory failure as seen in human (2).

Dexmedetomidine is widely used during the perioperative period due to its sedative, analgesic, sympatholytic, and hemodynamic effects on the patients undergoing surgery or under critical care. In addition, dexmedetomidine has anti-inflammatory and antioxidative effects on vital organs, such as lung and kidney (39, 40). The inhibitory effect of dexmedetomidine on the bilirubin-induced cell cycle arrest of A549 cells indicated that dexmedetomidine alleviates the injury of lung alveoli, possibly through promoting epithelial cell proliferation and initiating prompt repair. Dexmedetomidine, similar to other α_2_ adrenoceptor agonists, has a structure bearing similarity to imidazoline, and activation of imidazoline receptors may also contribute to its antiapoptotic effect (41). Pretreatment with α_2_ adrenoceptor antagonist atipamezole did not abolish the effect of dexmedetomidine on bilirubin-induced cell cycle arrest of A549 cells, which may indicate that the antiapoptotic effect of dexmedetomidine was not only due to an α_2_ adrenoceptor-dependent signaling pathway.

The benefits of dexmedetomidine for patients with pulmonary injury in the operation room and ICU have been observed in clinical trials (42, 43). In this study, dexmedetomidine was given to CBDL rats IP daily during the experimental period attenuated the lung injury and prevented respiratory failure development in the CBDL rat (Fig. 7). In addition, dexmedetomidine significantly reduced the apoptosis of lung cells of CBDL rats, and these cells not only limited to alveolar epithelial cells but other types as well (Fig. 8). However, owing to its short action, dexmedetomidine is normally administered by a continuous IV infusion clinically, but a long-term infusion of dexmedetomidine can be not done in our study. Hence, the translational value of our study can be questionable. One can appreciate that this is always the case in a preclinical study. Nevertheless, our data clearly showed that dexmedetomidine could directly protect the lung injury in our model study because α_2_ adrenoceptors distribute widely including the cell line that was used in our study (data not shown), but as reported previously (44), the protection could also be due to its indirect effects of a decrease of sympathetic tone and an increase of vagal tone. Collectively, our work reported here could call further large animal study and clinical trials in this area of research.

In summary, we demonstrated that dexmedetomidine attenuated the bilirubin-induced injury of epithelial alveolar cells both in vitro and in vivo. Although further studies especially in a large animal model and clinical trials are needed to further validate the protective effects of dexmedetomidine on HPS, its inhibitory effect on cell apoptosis and promoting effect on cell survival and proliferation represent a promising anesthetic/sedative choice in treating the patients with chronic lung injury following severe liver disease perioperatively.

## ACKNOWLEDGMENTS

We thank Yazhou Wu from the Third Military Medical University, China, for his advices on statistical analysis and thank James J. Sun from Cambridge University, United Kingdom, for his critical comment during manuscript preparation.
